# Treatise on the Ear

**Published:** 1840-07

**Authors:** 


ART. III.—*Treatise on the Ear; including its Anatomy, Physiology, and Pathology: for which the Author obtained a Gold Medal in the University of Edinburgh.* By JOSEPH WILLIAMS, M.D.—*London,* 1840. 8vo, pp. 255, and 6 Plates.

THE pathological is the only part of this treatise which merits any consideration. It is to be remarked that the acuter diseases of the ear, though the least frequent are not the least understood. The nature and treatment of habitual deafness are what we are most ignorant of; and cases of this kind unfortunately make up the great bulk of those which come under the practitioner's notice. We are unwilling to despair of an extension of our knowledge on the subject, but we are satisfied that hope can be reasonably entertained only when the well or ill founded nature of any particular view is closely scrutinized, and proved by an appeal not to assertion but to well-observed facts.

If our author had approached his subject in some such spirit as this, the talent which he undoubtedly displays would have enabled him to produce a treatise more worthy of approbation. He has indeed judiciously enough discussed the subject of the acuter diseases of the ear, but in treating of its less generally understood affections he has too frequently mistaken assertion for argument, and has even been occasionally forgetful enough to think to strengthen his own by coupling with them the equally vague assertions of others, in order to refute conclusions founded on well-directed observation and close reasoning. This is particularly exemplified in several flings he essays against Dr. Kramer's work. There are weak points in that work, on several of which we have on former occasions animadverted; but we must tell Dr. Williams that he has failed to adduce observations to invalidate what in it he objects to; and in his attempts to prove inconclusiveness of induction on the part of the author, he betrays, to say the least of it, an over-hastiness to blame, and no great knowledge of what he is writing of.

